# Real-time visualization of conformational changes within single MloK1 cyclic nucleotide-modulated channels

**DOI:** 10.1038/ncomms12789

**Published:** 2016-09-20

**Authors:** Martina Rangl, Atsushi Miyagi, Julia Kowal, Henning Stahlberg, Crina M. Nimigean, Simon Scheuring

**Affiliations:** 1 INSERM U1006, Aix-Marseille Université, Parc Scientifique et Technologique de Luminy, 163 Avenue de Luminy, Marseille 13009, France; 2 Center for Cellular Imaging and NanoAnalytics, Biozentrum, University of Basel, Mattenstrasse 26, Basel CH-4058, Switzerland; 3 Departments of Anesthesiology, Physiology and Biophysics, and Biochemistry, Weill Cornell Medical College, 1300 York Avenue, New York, New York 10065, USA

## Abstract

Eukaryotic cyclic nucleotide-modulated (CNM) ion channels perform various physiological roles by opening in response to cyclic nucleotides binding to a specialized cyclic nucleotide-binding domain. Despite progress in structure-function analysis, the conformational rearrangements underlying the gating of these channels are still unknown. Here, we image ligand-induced conformational changes in single CNM channels from *Mesorhizobium loti* (MloK1) in real-time, using high-speed atomic force microscopy. In the presence of cAMP, most channels are in a stable conformation, but a few molecules dynamically switch back and forth (blink) between at least two conformations with different heights. Upon cAMP depletion, more channels start blinking, with blinking heights increasing over time, suggestive of slow, progressive loss of ligands from the tetramer. We propose that during gating, MloK1 transitions from a set of mobile conformations in the absence to a stable conformation in the presence of ligand and that these conformations are central for gating the pore.

## Abstract (web summary)

Some ion channels are modulated by binding of cyclic nucleotide to a cyclic nucleotide-binding domain. Here, the authors use high-speed atomic force microscopy to directly monitor the conformational changes induced by ligand binding to a cyclic nucleotide-modulated channel from *Mesorhizobium loti*.

## Graphical abstract

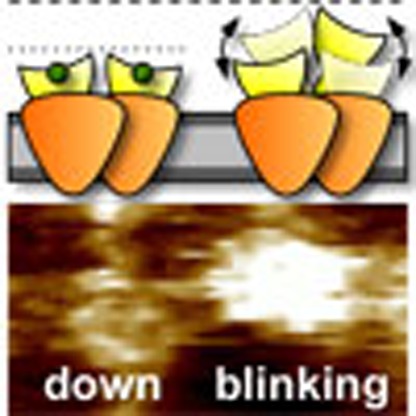

Ion channels are integral membrane proteins facilitating ion flux across cell membranes, thereby regulating signal pathways for physiological processes^1^. The activity of ion channels can be controlled by stimuli such as voltage, temperature, pH, mechanical stress and small signalling molecules such as Ca^2+^ and cyclic nucleotides^2^. Cyclic nucleotide-modulated (CNM) ion channels are key players throughout the entire nervous system as they regulate certain modes of signal transduction^3^,^4^ and play a role in neuronal excitability in brain and pacemaking in heart cells^5^,^6^. They detect levels of intracellular cyclic AMP or GMP (cAMP or cGMP) by direct binding to a specialized cyclic nucleotide-binding domain (CNBD). This chemical information, that is, ligand binding, is translated into an electrical response by opening of the channel pore allowing ion flow across the membrane.

Members of the family include the cyclic nucleotide-gated (CNG) and hyperpolarization-activated and cyclic nucleotide-gated (HCN) channels^5^,^7^. Both channel subfamilies belong to the S4 superfamily of voltage-gated cation channels that form tetramers whose subunits are aligned around a central pore. Each subunit consists of six transmembrane helices. The first four form the voltage sensor (S1–S4) and the last two form the pore (S5–S6) (Fig. 1a). The CNBD is C-terminally connected to S6 via a C-linker, a highly conserved domain within the family. CNG and HCN channels have been studied extensively with electrophysiological methods, while structural approaches were not very successful so far.

A prokaryotic homologue from the bacterium *Mesorhizobium loti*, MloK1, proved to be the best platform to study the structure-function relationship of CNM channels^8^. MloK1 is the only CNM channel for which an X-ray structure of the transmembrane region is available^9^. MloK1 also has X-ray and nuclear magnetic resonance structures of both liganded and unliganded isolated CNBDs, respectively^10^,^11^,^12^. Electron microscopy (EM) studies of full-length MloK1 (with and without ligand) as single particles and in two-dimensional (2D) crystals resulted in three-dimensional structures at 7–10 Å resolution^13^,^14^,^15^ that have been used for atomic modelling (Fig. 1b,c). A gating model was proposed, where the binding of ligand causes a movement of the CNBDs towards the membrane^15^. A similar movement, albeit larger in amplitude, was observed by conventional atomic force microscopy (AFM)^15^,^16^. All these techniques provided static views of these channels: full-length channels at medium resolution, and channel fragments at angstrom resolution.

We used high-speed AFM (HS-AFM)^17^ to explore the dynamics of MloK1 channels during gating and to study in real-time and with high resolution the conformational changes of MloK1 upon ligand binding/unbinding. HS-AFM allows the observation of single molecules with high lateral and temporal resolution (∼1 nm and ∼100 ms, respectively) under near-physiological conditions (that is, in buffer solution, at ambient temperature and pressure, without labelling, staining or fixing procedures) (Fig. 1d), ideal for monitoring conformational changes without the need for large fluorescent labels that may prevent free protein motion^18^,^19^,^20^,^21^. We exploited the large difference in protrusion heights between liganded and unliganded MloK1 channels, previously also detected by conventional AFM^15^,^16^, to monitor by HS-AFM in real time the dynamics of single MloK1 channels upon ligand depletion. We show here that MloK1 channels, as well as individual subunits within a tetramer, can switch back and forth (blink) between conformational states with different heights protruding from the membrane. At saturating cAMP, where most channels are fully liganded, the molecules are mainly in a down-state with low height, close to the membrane, and the incidence of blinking is low. Upon lowering the cAMP concentration, the number and height of blinking molecules gradually increase over time. Hence, we reasoned that the blinking process that we were able to capture using HS-AFM is specific to the unliganded CNBDs within a tetramer. The gradually increasing heights correspond to the gradually increasing number of unliganded, and hence blinking, subunits within the tetramer, during ligand depletion. We propose that upon ligand binding the channel switches from a set of flexible conformations created by the highly dynamic unliganded CNBDs to an ordered conformation with stable, liganded CNBDs, and that these conformations are important for gating.

## Results

### MloK1 channels display blinking behaviour

**Figure 1:**
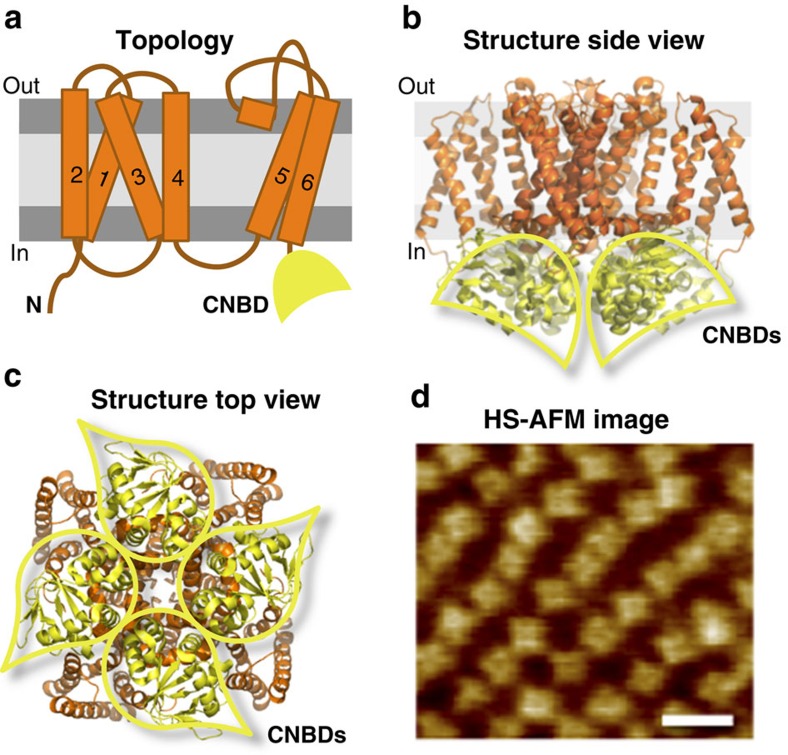
Architecture of MloK1. (**a**) Topology of one subunit with six transmembrane segments (orange); the C-terminus contains the CNBD (yellow). The voltage sensor (S1–4) and the pore (S5–6) are in the transmembrane region. Atomic model based on the cryo-EM MloK1 structure (PDB 4CHV, ref. 15), in (**b**) side and (**c**) top views, respectively. The CNBDs arrange in a wind mill-like fashion (yellow outlines). (**d**) High-resolution HS-AFM topograph of MloK1 in 2D crystals in presence of 100 μM cAMP. Scale bar: 15 nm. Full colour scale: 2 nm.

**Figure 2:**
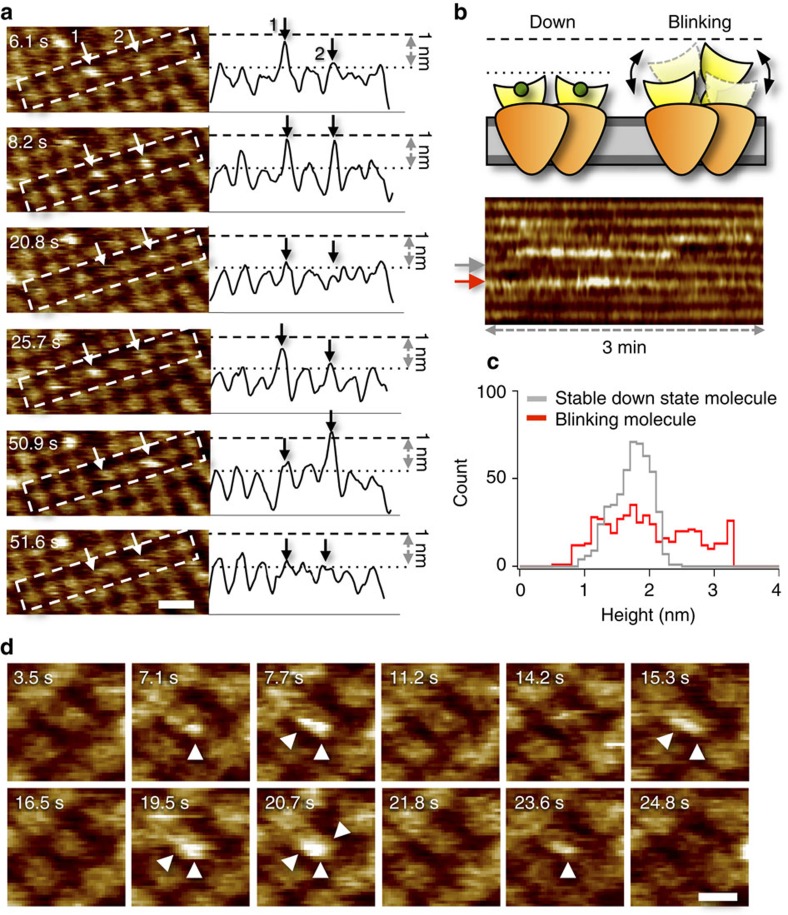
MloK1 dynamically changes protrusion height during HS-AFM imaging. (**a**) Representative HS-AFM movie frames of the same MloK1-containing membrane. Arrows 1 and 2 highlight two individual molecules continuously blinking between two heights. On the right are the corresponding height profiles along the centre axis of the outlines in each frame on the left. During blinking the CNBDs undergo a height change of ∼1 nm. Scale bar: 20 nm. Full colour scale: 2.5 nm. (**b**) Cartoon of the blinking process: unliganded CNBDs alternate between down- and up-states. The kymograph (lower) plots the height evolution over 3 min for the eight MloK1 molecules highlighted in **a**. Colour display is identical. (**c**) Height histograms of the non-blinking (grey) and blinking (red) molecule from the kymograph in **b** (arrows). While the non-blinking channels show a one-peak distribution, the blinking channels display a broad height histogram. (**d**) High-resolution HS-AFM image frames showing an example of individual blinking subunits (arrows) within an MloK1 tetramer. Scale bar: 10 nm. Full colour scale: 2 nm. Buffer contains 10 nM cAMP for **a**–**c**, and 100 μM cAMP for **d**.

HS-AFM was used to image dynamics of ligand-induced structural changes in MloK1. For this, 2D crystals of wild-type MloK1, grown in the presence of cAMP^15^, were used. Large membranes with densely packed channels were imaged in 100 μM cAMP. High-resolution movies revealed individual channels as windmill-like structures formed by four clearly distinguishable CNBDs emerging 1.6±0.1 nm from the membrane (Fig. 1d). The structure and dimensions were in agreement with results obtained by conventional AFM^15^ and earlier work^13^; however, the fast HS-AFM imaging acquisition in the sub-second range (down to 80 ms) revealed that about 10% of the molecules dynamically switched between two conformations of different heights. These changes would not have been observable with conventional AFM techniques due to their low time resolution. A representative membrane area shows several individual molecules blinking (Fig. 2a, left; from an experiment performed in 10 nM cAMP) and switching back and forth between two height levels over time (Fig. 2a, right). In an attempt to measure the dwell-times in the up- and down-states, we monitored blinking molecules with scan rates between 80–800 ms per frame. However, blinking molecules had a height-change rate of typically 1–2 frames even at the fastest scan rates, suggesting that the dwell-times of the blinking states are faster than the time resolution of ∼10 s^−1^. This was the case over a wide range of cAMP concentrations (10 nM–1 mM).

The blinking (Fig. 2b, top) can be captured in kymographs over extended imaging periods, illustrating the reversible nature of this conformational change in the channels (Fig. 2b, bottom). The blinking state illustrated here is reminiscent of the ill-defined unliganded MloK1 channels, previously reported by conventional AFM^15^,^16^, but its height is dynamically changing. The up-down height switch of individual molecules (Fig. 2b) is reflected in a drastic broadening of the height distribution of blinking molecules, which was used as criterion for discerning blinking from non-blinking channels (Fig. 2c). As the blinking CNBDs fluctuate in height, high-resolution imaging at the submolecular level is hard to achieve. However, in rare cases, we were able to visualize blinking of single CNBDs in a channel tetramer (Fig. 2d and Supplementary Movie 1) suggesting that each subunit can undergo these conformational changes individually.

### The number of blinking MloK1 channels increases in low cAMP

Since MloK1 CNBDs imaged by AFM in ligand-free conditions display an increased height, which is in agreement with the unliganded EM structure showing a detachment of the CNBD from the membrane^15^, we hypothesized that the observed back and forth height changes seen in the blinking molecules are related to ligand dissociation from the channels. In this case, lowering the cAMP concentration should lead to more channels losing their ligands and an increase in the number of blinking molecules. Hence, we analysed MloK1 blinking at 10 nM cAMP, a concentration about ten times lower than the reported affinity (*K*_D_ ∼80 nM; ref. 22), and where most molecules are expected to lose their ligands. We chose 10 nM instead of 0 nM cAMP because we wished to preserve a few molecules in an ordered, liganded conformation in the imaged membrane, as an important technical quality control.

**Figure 3:**
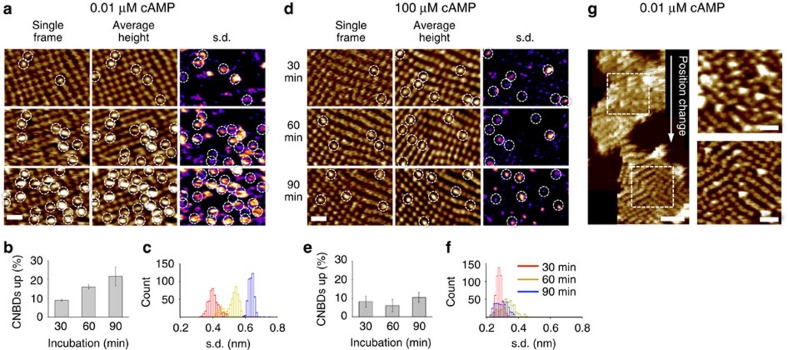
The number of blinking MloK1 channels increases over time at low ligand concentration. (**a**) Representative membrane imaged after 30 (upper), 60 (centre) and 90 (lower) minutes in 10 nM cAMP. For each condition, a single HS-AFM frame (left), the average height (middle) and the s.d. map (right) of the complete movie are shown. Blinking channels are highlighted by circles. While the single frame only shows MloK1s with elevated CNBDs at a given time, the average height-map displays all molecules with elevated heights throughout the movie, and the s.d.-map highlights the pixels changing over time, a hallmark of blinking. (**b**) The percentage of blinking channels in 10 nM cAMP plotted as a function of incubation time (mean±s.d.). (**c**) Histograms of the s.d. of all pixel values in each movie frame from a membrane area of 12,750 nm^2^ (15,000 pixels) and ∼500 frames at 10 nM cAMP after 30 (red), 60 (yellow) and 90 (blue) minutes. An increase in the s.d. reflects increased blinking. (**d**–**f**) as described in **a**–**c**, but in the presence of 100 μM cAMP. In contrast to the low-cAMP condition, the number and intensity of blinking molecules stays virtually identical over time. (**g**) The height increase of the CNBD in low cAMP requires an input of energy provided by the AFM tip. The imaged membrane area shows an increasing number of blinking molecules (upper), while most MloK channels are in cAMP-bound conformation in the untouched membrane area (lower). The full colour scales and scale bars are identical for all AFM images and correspond to 3 nm (*z*) and 30 nm (*x*/*y*).

As reported previously, complete removal of cAMP from the CNBDs of full-length MloK1 in dense reconstitutions is a slow process (hours to days) reflecting a slow off-rate of the ligand from the binding site^11^,^15^,^22^ Accordingly, the molecules were repeatedly imaged by HS-AFM over 90 min in 10 nM cAMP to capture their development over time (Fig. 3a and Supplementary Movie 2). After 30 min of incubation in low cAMP, the majority of the MloK1 molecules observed were still in the down-state, presumably cAMP-bound, and only 9±1% of the molecules were blinking (Fig. 3a, upper), and therefore no difference was observed from the high-cAMP condition. However, after re-imaging the same patch after 60′ (Fig. 3a, middle) and then 90′ (Fig. 3a, lower panel), the number of blinking MloK1 molecules steadily increased from ∼10 to ∼25% (Fig. 3b). This is illustrated in time-averaged topography maps (Fig. 3a, centre panels), in which blinking molecules appear as brighter, often blurry spots. The blinking is even more apparent in s.d. maps over all movie frames: the brighter the area, the larger the height variability, indicating blinking (Fig. 3a, right). This increase in height variability (blinking) over time in the imaged area is highlighted in the s.d. histograms (Fig. 3c).

The increase in the number of blinking molecules in low cAMP is not due to a systematic error from the scanning process or sample deterioration by the AFM tip because membranes imaged in 100 μM cAMP over the same period of time (Fig. 3d and Supplementary Movie 3) did not change in either the number (compare Fig. 3e with Fig. 3b and Supplementary Movie 2 with Supplementary Movie 3) or height variability (compare Fig. 3f with Fig. 3c) of blinking molecules. In addition, the height of the non-blinking molecules in the vicinity of blinking ones remained constant over time. This strongly indicated that the increase in blinking over time was related to a gradual loss of cAMP from the CNBD binding pocket at low ligand concentration.

Interestingly, the exit of ligand from the binding pockets appeared to be helped to some extent by the energy input of the HS-AFM tip. Molecules in membrane patches that were not imaged, maintained their low heights and windmill-like appearance after 2 h incubation in 10 nM cAMP (Fig. 3g), while repeatedly imaging a different region of the same membrane after the same amount of time in the same conditions led to an increase in molecule heights and blinking, as shown above (Fig. 3). Again, this did not happen in high-cAMP conditions, directly correlating the increase in blinking upon tip interaction with loss of cAMP (Fig. 3d).

### Binding of cAMP to ligand-free MloK1 channels is slow

**Figure 4:**
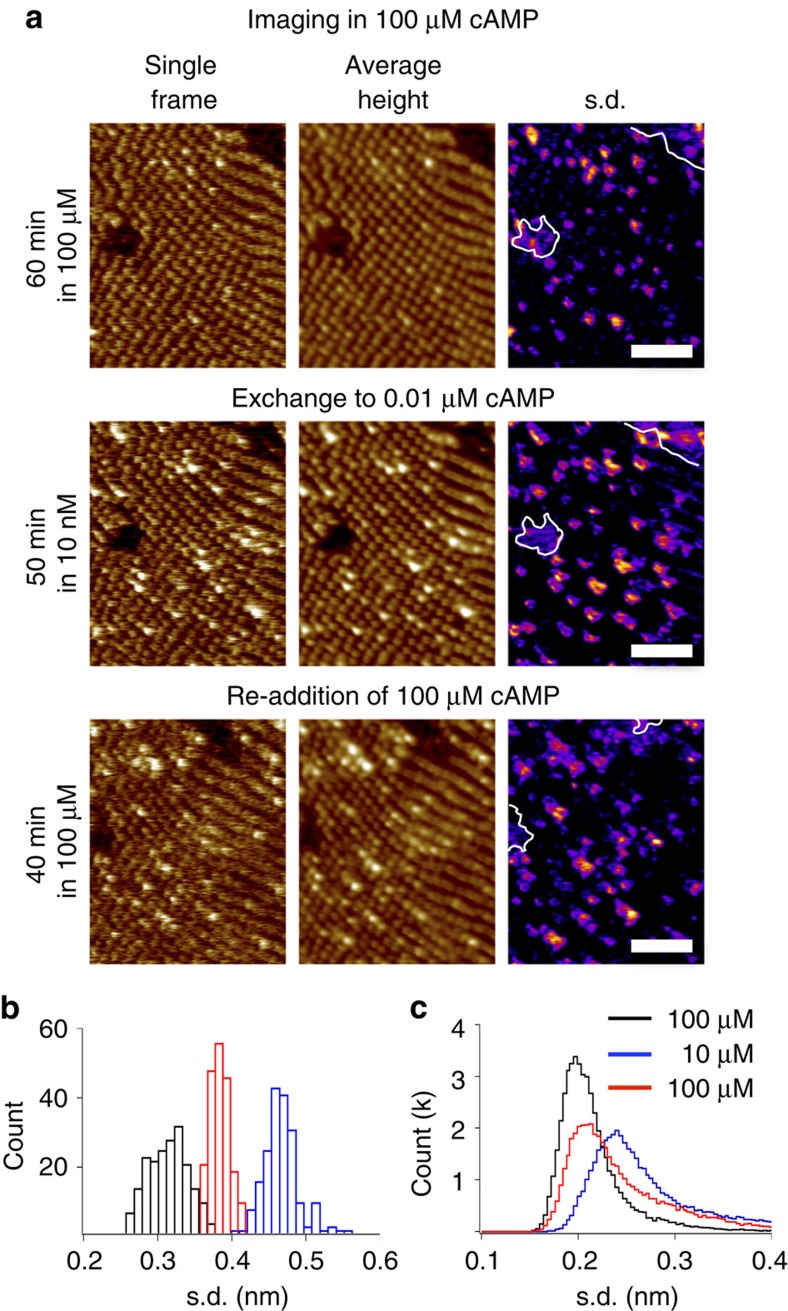
Reversibility of CNBD blinking. (**a**) An identical membrane imaged in presence of 100 μM cAMP (upper), then in 10 nM cAMP (middle), and after re-addition of 100 μM cAMP (lower). Single HS-AFM frames (left), average height topographies (centre) and the s.d. maps (right) of the movies are shown. Scale bar: 50 nm. Full colour scale: 3 nm. (**b**) Histograms of the s.d. of each original movie frame (*n*=200) consisting of 30,000 pixel at 100 μM cAMP (black), 10 nM cAMP (blue), and after re-introduction to 100 μM cAMP (red). An increase in the s.d. distributions reflects increasing height distributions. (**c**) Histograms of the s.d. maps of the movies (**a**, right). The more blinking molecules, the higher the height variability.

To understand the association process of cAMP to MloK1 channels that have lost the ligand, we monitored an identical membrane over ∼4 h at different ligand concentrations. We first imaged the channels in presence of 100 μM cAMP for ∼60 min (Fig. 4a, upper), after which the ligand was washed out, using a buffer exchange system^23^, and the channels were imaged several times in 10 nM cAMP for an additional hour. We stopped imaging in low-cAMP before all molecules lost their ligand, as evidenced by lack of blinking and maintained low height in at least half of the molecules (Fig. 4a, middle). At this point, we reintroduced cAMP (100 μM) into the buffer. The reintroduction of cAMP stopped the increase in the number of blinking molecules, lead to a decrease in blinking activity and a few molecules even reverted to their initial lower height (Fig. 4a, bottom panel). The s.d. of height values of each individual frame (Fig. 4b) and the pixel-by-pixel s.d. along the time axis (Fig. 4c) indicate that reintroduction of cAMP led to fewer molecules blinking and to lower heights. However, full recovery of the windmill-like structures of the molecules could not be achieved even after 1 h. This suggests that recovery, which includes ligand binding to all four subunits and rearrangement to the windmill structure, is a slow process, of the same order as the ligand unbinding from the CNBD^15^.

### MloK1 channels blink to higher heights over time in low cAMP

**Figure 5:**
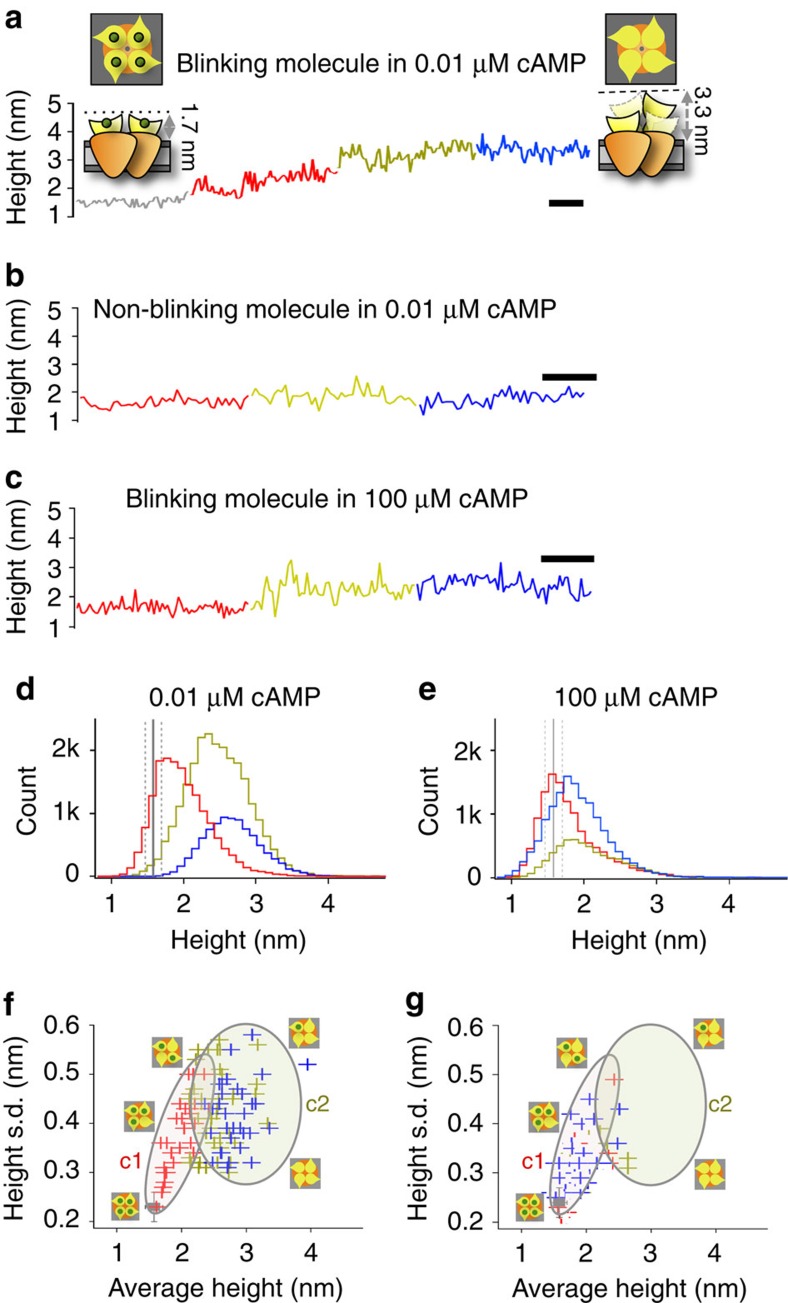
MloK1 blinking heights increase over time in low cAMP. (**a**) Height versus time representative trace of an individual channel after incubation for 30 (red), 60 (yellow) and 90 (blue) minutes in 10 nM cAMP. A non-blinking channel from the same patch is shown in grey. Over time the molecule traverses several height levels. Cartoons representing liganded and unliganded MloK1 are shown. (**b**) Height versus time representative trace of a non-blinking channel after 30, 60 and 90 min in 10 nM cAMP (same colour scheme as in **a**). In contrast to the blinking molecule, no progressive height increase could be detected. (**c**) Height versus time representative trace of a blinking channel at 100 μM cAMP after 30, 60 and 90 min (same colour scheme as in **a**). The molecule starts blinking at the second scanning round (60 min) and keeps blinking at the same height level throughout the entire experiment. Scale bar: 10 s (**a**–**c**). (**d**,**e**) Histograms of MloK1 heights after 30, 60 and 90 min incubation in 10 nM cAMP (**d**) or100μM cAMP (**e**), respectively. Colour code is as in **a**. As a reference, the average height (solid line) and s.d. (dashed line) of non-blinking down-state molecules (grey, *n*=18 (10 nM cAMP) and *n*=15 (100 μM cAMP) molecules) are shown. (**f**) Correlation between average height and height s.d. for each molecule at 10 nM cAMP. Colour code as above. Each cross in the distribution is the average height of a single molecule plotted against the s.d. from all 500 frames. Mean±s.d. of non-blinking molecules (*n*=18) are shown as a grey square. Blinking molecules pass through different height regimes (clusters c1 and c2, outlined by large circles). (**g**) correlation between average height and height s.d., at 100 μM cAMP. Mean±s.d. of non-blinking molecules (*n*=15) is shown as a grey square.

If the blinking process in a channel is due to ligand loss from individual CNBDs, then the height to which channels blink will depend on how many CNBDs within the tetramer have lost their ligand, because in overview images the HS-AFM measures an average height over all four CNBDs. For instance, if only one CNBD in a tetramer would have lost its ligand, then the overall blinking height of that channel would be lower than if all four CNBDs would have lost the ligand and switch between up and down states. We thus performed a height analysis of individual blinking molecules. For this, single molecules were tracked within subsequent movies at different incubation times and their heights were compared. The non-blinking, stable molecules, showed an average protrusion height of 1.6±0.2 nm and fluctuated just slightly after 30 min incubation in 10 nM cAMP (Fig. 5a, grey trace). In the same conditions, the blinking molecules show larger fluctuations and reach a higher height level of 2.0±0.3 nm (illustrated by the molecule tracked in Fig. 5a, red trace). The identical blinking molecule tracked after 60′ and then 90′ displayed progressively higher heights of 2.5±0.4 nm and 2.8±0.4 nm, respectively (Fig. 5a, yellow and blue traces, respectively), where the blinking height reached a plateau. As a control, an identical non-blinking molecule in the same membrane area showed no height increases over time (1.6±0.4 nm after 60′ and 1.6±0.3 nm after 90′; Fig. 5b) highlighting that the above-described gradual height increase is specific to the subset of blinking molecules and is not a degradation of the whole membrane. As a second control, a similar analysis of the blinking molecules in 100 μM cAMP, revealed that similar prolonged incubations and scanning did not lead to the gradual increase in heights as observed in 10 nM cAMP (compare Fig. 5a,c).

Altogether, the gradual height increase in blinking molecules occurs only in the low ligand concentrations (Fig. 5d), and not when saturating cAMP is provided (Fig. 5e). Accordingly the gradual height increase is consistent with a progressive, one-by-one cAMP dissociation from individual CNBDs within a tetramer over time. Thus, in an ideal situation a direct classification of molecules with 0, 1, 2, 3 or 4 CNBDs unbound should be possible using a height threshold approach. However, in this case, the contribution of an individual subunit to the height increase is too small and the variability of each state is too large precluding such an analysis (see Fig. 5).

Hence, in order to correlate the height values with ligand loss, the average height for each blinking molecule (a measure of how many cAMP molecules have unbound from a tetramer) was plotted as a function of its s.d. (a measure of the ‘blinking activity') (Fig. 5f). We found that molecules displayed a trend where larger heights corresponded to increased blinking activity up to a height of ∼2.5 nm (Fig. 5f, cluster 1). Molecules with further height increase to ∼3.1 nm displayed high blinking activity (cluster 2). We interpret cluster 1 to represent mainly tetramers that lost one or two ligands, while the molecules in cluster 2 comprised channels that approached the fully unliganded state. The few blinking molecules in the movies acquired in 100 μM cAMP populated cluster 1 (Fig. 5g) suggesting that in saturating cAMP the majority of the CNBDs remain liganded. The detection of single CNBDs blinking independently within a tetramer (Fig. 2d and Supplementary Movie 1) lends strong support to the above-described hypothesis that the gradual height increase of the entire channel indeed represents a successive loss of ligands from each subunit in the tetramer. Interestingly, it appears that if one CNBD loses its ligand and starts blinking, then other subunits within the same tetramer are favoured to also lose their ligand and start blinking, suggesting that there is some crosstalk within the tetramer. This is evident from the multiple subunits blinking within a molecule in Fig. 2d, as well as in the trend of the height increase over time within the same molecules upon ligand depletion while neighbouring tetramers are not affected (Fig. 5a,d).

All of the above experiments predict that in the complete absence of ligands, MloK1 channels will blink continuously, as all of their CNBDs are now devoid of ligand. To investigate this, we imaged an MloK1 membrane patch that was dialyzed over 2 weeks in cAMP-free buffer, conditions shown previously to lead to the complete removal of cAMP from the sample^15^, and we indeed observed individual MloK1 channels blinking (Supplementary Fig. 1). We could not quantify this effect as the resolution of individual molecules in these conditions is low, as previously reported^15^,^16^. The alternating head-to-tail packing of the MloK1 tetrameric channels in the membranes leads to channel CNBDs facing the underlying mica support in the HS-AFM, so that the blinking of these molecules will result in instability of the entire membrane. This instability leads to the observed resolution loss in this experiment and explains the somewhat lower resolution of HS-AFM imaging under low cAMP conditions.

## Discussion

We captured the dynamics of the conformational changes of MloK1 channels upon cAMP unbinding using HS-AFM. As MloK1 loses ligands from its CNBDs, the molecule starts ‘blinking', that is, the topography height of the CNBDs is switching back and forth between at least two levels. With time, the process tends towards higher heights that correspond to an increasing number of ligands unbound from the CNBDs, until all ligands have dissociated and all four CNBDs within a tetramer are blinking. The fully liganded and unliganded MloK1 states have been previously characterized with EM and AFM^15^,^16^ by presenting static snapshots of supposedly two distinct states. Our real-time imaging study provides an analysis of the conformational changes undergone in MloK1 as single molecules transit between several height regimes in the process of losing all four ligands over time at sub-*K*_D_ cAMP concentration and show that the process is more complex than what a two-state model would propose.

The blinking process may represent a conformational change directly reflecting ligand binding and unbinding from a CNBD, where the bound state is represented by a down- and the unbound state by an up-conformation. Alternatively, it may represent an equilibrium process that occurs within each unliganded subunit where the subunit continuously switches between down- and up-conformations when the ligand is lost. Our results favour the latter explanation for the following reasons. First, in the presence of saturating ligand, the same molecule, and in some cases the same subunit, is observed to continuously blink over extended imaging periods. This is consistent with an unliganded molecule oscillating between conformations, as it is highly improbable that the same exact molecule continuously binds and unbinds ligand over an extended period of time. Second, the reported cAMP unbinding rate from the CNBD in the full-length MloK1 is about 0.2 s^−1^ on average^24^, which is much slower than the blinking frequency measured at our highest imaging rates (>10 s^−1^). Third, the blinking rates do not depend on the cAMP concentration, which they should if the on-rate depended on the ligand abundance in the environment. Fourth, if a blinking subunit is the result of unliganding its CNBD, then a fully unliganded channel should be a mobile unit consisting of four blinking subunits. In contrast, if each blinking event represented binding and unbinding of ligand, then a fully unliganded channel would have a non-blinking, stable and resolvable conformation with higher height. The former is supported by our findings: the measured height s.d. is high for unliganded channels and fully unliganded channels remain unresolved and blink.

A variable channel height caused by continuous subunit blinking in unliganded CNBDs offers an explanation for the discrepancy between the amplitudes of the CNBD movements upon ligand removal observed with EM and conventional AFM^15^,^16^. In the EM structure, the CNBDs shifted only 3 Å away from the membrane, while the AFM experiments suggested a height increase of ∼15 Å between the liganded and unliganded states. Since the EM structure is an average over hundreds of thousands of individual molecules in the 2D crystal, molecules with different heights would be averaged together in the crystal to yield an average height lower than the maximum height observed with direct single molecule HS-AFM measurements.

The imaging resolution decreases dramatically as liganded channels become unliganded. The loss of resolution precluded in most cases the visualization of individual subunits in the tetramer upon ligand dissociation, consistent with the previously reported flexibility of unliganded CNBDs^9^,^10^,^11^,^12^. However, in the highest resolution HS-AFM movies available, blinking of individual subunits within an MloK1 tetramer is observed, strongly supporting the hypothesis that the height increase upon ligand removal corresponds to individual subunits within the tetramer losing their ligands in succession. The gradual increase in measured heights indicates that the ligands unbind successively in low-cAMP condition, suggesting low cooperativity in ligand unbinding, as it has been previously suggested^22^,^25^. The observation of individual subunits within a tetramer blinking independently of the rest of the tetramer over extended periods of time corroborates this interpretation. However, the observation that blinking in one subunit favours blinking in a neighbouring subunit within the same channel suggests that there is some crosstalk between subunits within a tetramer such that unbinding of cAMP from one CNBD tends to increase the probability of ligand loss in the other CNBDs of the same molecule.

Multiple studies, as well as our work, report that the cAMP-CNBD interaction is so strong in MloK1 that only long dialysis (over several days) and lower-affinity CNBD mutants allow cAMP removal^11^,^15^,^22^. In contrast, kinetic rates significantly faster (milliseconds to seconds) were shown in stopped-flow experiments using detergent-solubilized MloK1^24^. In our experiments however, the channels are in a lipid bilayer and densely packed in 2D crystals, which may account for the differences in the rates. Furthermore, within an immobilized membrane adsorbed to the support in the HS-AFM, ligands unbinding from CNBDs may well rebind to it before being able to escape into bulk, leading to an apparent decrease in the off-rate.

We found that HS-AFM imaging can favour cAMP removal from the CNBD and we hypothesized that the HS-AFM tip tapping lowers slightly the energy barrier for ligand dissociation so that a few more molecules on average lose their ligand. To estimate the required external energy, we considered typical HS-AFM conditions (that is, 100 pN nm^−1^ spring constant, 600 kHz resonance frequency, 1 nm free oscillation amplitude, and 0.9 nm setpoint amplitude) yielding in an impact force on the sample in the 10^−18^ Ns range per tap, which corresponds to about 1–2 *k*_B_*T* (refs 18, 26). It is noteworthy that under the same imaging conditions but with saturating cAMP, the cAMP-bound CNBDs can be imaged faithfully over extended periods of time, suggesting that the tip does not damage the sample. The same holds true for numerous other proteins we and others have studied before^18^,^19^,^20^,^21^,^27^,^28^. Alternatively, the stirring of the solution layers around the protein through the HS-AFM tip scanning might account for a faster ligand-release rate than molecules that are not scanned.

Why is the ligand released in low cAMP conditions upon tapping and not in high cAMP? In the presence of ligand, the cAMP released from the binding pocket upon AFM tip tapping is immediately replaced and the probability of going into a cAMP-free state is low. At 10 nM cAMP, the binding pocket remains empty and the subunit starts blinking. This predicts that reintroduction of ligand should fill the empty CNBDs and recover the low height, low blinking, and windmill-like appearance of the molecules, which does not happen in our experiments. We propose that instead of a simple bimolecular reaction where the CNBD binds ligand, the CNBDs in the tetramer can exist in two conformations, with an open lid, allowing free access of ligand to the binding pocket, or with a closed lid, sterically preventing ligand entrance or exit (Supplementary Fig. 2a). In support of this model, in the existing crystal structures the CNBD binding pocket is formed by a β-roll and a phosphate-binding cassette covered by an α-helical lid, the C-helix, which has been shown to adopt different conformations (Supplementary Fig. 2b)^10^,^11^,^12^. We propose that the lid-closed conformations, both ligand-bound and ligand-free, have long dwell-times in the tetramer so that ligand exit and entry are sterically hindered (thick arrows in Supplementary Fig. 2a). Thus, it is likely that during our experiments, we mostly image these long-lived closed-lid conformations, both liganded and unliganded. This steric hindrance can explain why it takes days to empty the MloK1 CNBDs of ligand when bathed in low ligand concentration^15^, and why supplying ligand to ligand-free channels does not lead to immediate recovery (on the time scale of hours for our experiment). We propose that tapping with the AFM tip provides energy to favour the opening of the lid and thus speeds up this rate-limiting step in the channel, allowing faster emptying of the CNBDs (minutes as opposed to days). The model also offers an explanation for the rare blinking events that nevertheless occur repeatedly within the same tetramer in the presence of cAMP; in the rare events that the lid closes without a ligand bound in high-cAMP conditions, it starts blinking, and the probability for re-binding is low since the lid-opening is the rate-limiting step.

All our observations indicate that the transition from a fully liganded to an unliganded MloK1 channel occurs gradually and can be described as following: in the fully liganded channel, the CNBDs are arranged close to the membrane and likely make contact with the membrane and/or the voltage sensor domain^15^, giving it the observed ordered windmill-like structure we image in high-cAMP. If only three binding-sites are occupied with cAMP, the MloK1 channel starts to blink as the empty CNBD starts fluctuating between up- and down-conformations. During extended immersion in low-cAMP, the tetramer then loses gradually all four ligands from the binding pockets, all CNBDs start blinking independently leading to a highly dynamic tetramer (Supplementary Movie 4).

In conclusion, we show that MloK1 channels can blink in both presence and absence of cAMP. The blinking and accompanying height increase is cAMP-dependent and associated with structural changes occurring in the CNBDs upon ligand unbinding. This transition from a channel with highly mobile unliganded CNBDs to an ordered, windmill-like arrangement of CNBDs upon ligand binding has likely a major role in the gating of MloK1 channels and of other CNG channels within the same family. We demonstrate here the unique capability of HS-AFM to study dynamic processes at the single unlabelled membrane protein level for the characterization of protein–ligand interactions and conformational changes that would be difficult to impossible to investigate with any other technique.

## Methods

### MloK1 expression and purification

Full-length MloK1 channels were expressed and purified as previously described^8^,^13^,^15^. Briefly, a C-terminal hexahistidine-tagged MloK1 construct in a pASK90 expression vector was transformed in *Escherichia coli* BL21(DE3) cells (New England Biolabs) and protein expression was induced by addition of 0.2 mg l^−1^ anhydrotetracycline for 2 h. The cells were harvested, sonicated and the membranes solubilized with 1.2% *n*-decyl-maltoside (DM, Anatrace) for 2.5 h at 4 °C. The solubilization buffer (SB) had 295 mM NaCl, 5 mM KCl, 20 mM Tris-HCl pH 8.0, 0.2 mM cAMP. During sonication 10% glycerol, 1 mM phenylmethylsulphonyl fluoride were added. The extract was spun down at 37,000*g* and the supernatant was applied to a Co^2+^ affinity column, which was washed with buffer SB with 0.2% DM and 40 mM imidazole. The protein was eluted with buffer SB with 0.2% DM and 500 mM imidazole. Detergent-solubilized MloK1 was mixed with *E. coli* polar lipid extract (Avanti Polar Lipids) at a lipid-to-protein ratio of 0.8–1.0 and dialyzed against detergent-free buffer (20 mM KCl, 20 mM Tris-HCl pH 7.6, 1 mM BaCl_2_, 1 mM EDTA, 0.2 mM cAMP) for 2D-crystallization in dialysis buttons for 5–10 days (in buffer with cAMP ligand) at 37 °C (the dialysis buffer was exchanged every other day). After crystallization and before HS-AFM analysis, the C-terminal hexahistidine-tagged was cleaved for 2–3 h at 20 °C with 200 NIH units of thrombin (Sigma) per mg of protein. To stop the reaction, 0.1 mM Pefabloc (Sigma) was added and sample was washed three times with crystallization buffer. Supernatant was removed after the 2D crystals had sedimented and fresh crystallization buffer added.

### HS-AFM imaging

A 1mm diameter muscovite mica plate was glued on a HS-AFM glass rod sample holder and mounted on the scanner. MloK1 2D crystals were adsorbed on freshly cleaved mica for 20 min. Subsequently the sample was rinsed with imaging buffer (10 mM Tris, pH 7.4, 150 mM KCl) containing 100 μM cAMP. To change the cAMP concentration, the buffer solution in the imaging pool was washed with five volumes of imaging buffer containing 10 nM cAMP, using an integrated constant-pressure and constant-flow pump system^23^ (Harvard Instruments, USA). HS-AFM^17^,^18^ (RIBM, Japan) was operated in oscillating mode, equipped with ultra short cantilevers of 8 μm in length (USC, NanoWorld, Switzerland) with a spring constant of 0.15 N m^−1^, a resonance frequency of ∼600 kHz and a quality factor of ∼1.5 in buffer. The applied force to the sample was minimized by adjusting the free amplitude to ∼10 Å and the imaging amplitude setpoint to ∼90% (∼9 Å) of the free amplitude (the force *F* can be estimated following *F*={*k*_*c*_[(1−α)·(*A*_0_^ 2^−*A*_S_^ 2^)]^1/2^}/*Q*_*c*_, where α is the ratio of amplitude reduction by frequency shift to total amplitude reduction (typically α=0.5), *k*_*c*_=0.15 N m^−1^ is the cantilever spring constant, and *Q*_*c*_=1.5 is the cantilever quality factor in liquid). Membrane areas of 150 × 150 nm^2^ to 250 × 250 nm^2^ were imaged, using 200 × 200 or 300 × 300 pixels per frame, respectively, with scan speeds between 300 and 600 ms per frame.

### Data analysis

HS-AFM images were first-order flattened and contrast adjusted using laboratory-made routines in Igor Pro software (WaveMetrics, Lake Oswego, OR, USA). The movies were then drift corrected, by means of frame-to-frame cross-correlation, using a lab-made image analysis software plug-in for ImageJ^29^,^30^. Average topography and s.d. maps of the movies were calculated using the standard measurement tools in ImageJ. Protrusion heights (subtracting the minimum pixel value from the maximum pixel value), height averages and s.d.s were calculated of each single molecule, in a box of 20 × 20 nm^2^ around the centre of weight of the molecule in each frame. For each condition 2 or more membrane patches were analysed with movies consisting of∼500 frames each.

### Data availability

The data that support the findings of this study are available from the corresponding author upon request.

## Additional information

**How to cite this article:** Rangl, M. *et al*. Real-time visualization of conformational changes within single MloK1 cyclic nucleotide-modulated channels. *Nat. Commun.* 7:12789 doi: 10.1038/ncomms12789 (2016).

## Supplementary Material

Supplementary Information Supplementary Figures 1-2 and Supplementary References.

Supplementary Movie 1 High-resolution HS-AFM movie of individual blinking CNBDs. Within a single MloK1 tetramer individual CNBDs can blink independently. HS-AFM movie acquired at 540ms per frame. Full frame size: 30x30nm. Full color scale: 2nm.

Supplementary Movie 2 HS-AFM movies of MoK1 membrane in 10nM cAMP imaged after 30, 60 and 90 minutes incubation time. At low cAMP concentration the number of blinking MloK channels is gradually increasing over time. HS-AFM movies were acquired at 700ms per frame. Full frame sizes: 150x100nm. Full color scales: 3nm.

Supplementary Movie 3 HS-AFM movies of MoK1 membrane in 100uM cAMP imaged after 30, 60 and 90 minutes minutes incubation time. At saturating cAMP concentration MloK1 channels can be stably imaged and the number and intensity of blinking molecules stays virtually identical over time. HS-AFM movies were acquired at 540ms per frame. Full frame sizes: 150x100nm. Full color scales: 3nm.

Supplementary Movie 4 Schematic representation of cAMP dissociation model from CNBDs in the tetramer. Upon cAMP dissociation the unliganded, individual CNBDs start blinking. Transmembrane domains are represented in orange, CNBDs in yellow, and cAMP as green circles.
