# Supplementary material for: Real-time visualization of conformational changes within single MloK1 cyclic nucleotide-modulated channels
Source: Nat Commun. 2016 Sep 20;7:12789. doi: 10.1038/ncomms12789 (PMC5034309; doi:10.1038/ncomms12789)
Supplement: Supplementary Information — Supplementary Figures 1-2 and Supplementary References. [file ncomms12789-s1.pdf]

Supplementary Figure 1

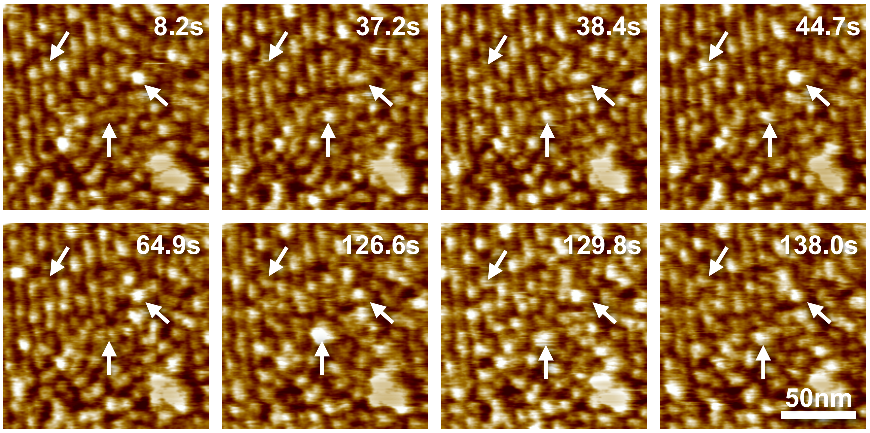

Supplementary Figure 1: Blinking CNBDs in completely ligand-free condition. *Representative HS-AFM movie frames of an MloK1 membrane subjected to a two-week long dialysis in cAMP-free buffer, as described in Kowal et al., 2014, ref.* [1]. *Arrows highlight some molecules that continuously change height. Scale bar: 50nm.*

Supplementary Figure 2

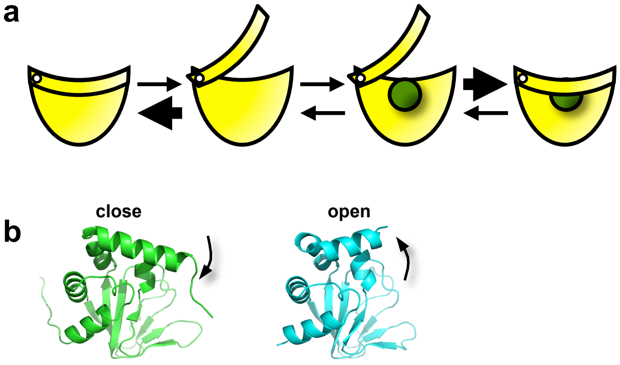

Supplementary Figure 2: Ligand binding model to CNBDs in the tetramer. ***a)*** *CNBDs (yellow) adopt at least 2 conformation in each state: (from left to right) unliganded-closed and -open lid, and liganded-open and -closed lid. Both closed-lid conformations are favored in the tetramer as represented by thick arrows. Ligand is represented by green sphere. Only one CNBD in the tetramer is depicted for simplicity. While the liganded states show a stable, membrane-close arrangement, the unliganded states appear dynamic and blinking.* ***b)*** *Structures of the CNBDs in closed (left, PDB 2KXL*[2]*) and open (right, PDB 3CO2*[3]*) lid conformations.*

Supplementary References

1 Kowal, J. *et al.* Ligand-induced structural changes in the cyclic nucleotide-modulated potassium channel MloK1. *Nat Commun* **5**, 3106, doi:ncomms4106 [pii] 10.1038/ncomms4106 (2014).

2 Schunke, S., Stoldt, M., Lecher, J., Kaupp, U. B. & Willbold, D. Structural insights into conformational changes of a cyclic nucleotide-binding domain in solution from Mesorhizobium loti K1 channel. *Proc Natl Acad Sci U S A* **108**, 6121-6126, doi:1015890108 [pii] 10.1073/pnas.1015890108 (2011).

3 Altieri, S. L. *et al.* Structural and energetic analysis of activation by a cyclic nucleotide binding domain. *J Mol Biol* **381**, 655-669, doi:S0022-2836(08)00698-0 [pii]10.1016/j.jmb.2008.06.011 (2008).
